# Spin-coated CZTS films prepared by two different precursor mixing regimes, at room temperature and at 150 °C

**DOI:** 10.1016/j.heliyon.2024.e25354

**Published:** 2024-01-28

**Authors:** R. Aliti, Y. Putthisigamany, P. Chelvanathan, M. Ristova

**Affiliations:** a Institute of Physics, Faculty of Natural Sciences and Mathematics, Ss. Cyril and Methodius University, Arhimedova 3, 1000, Skopje, North Macedonia; b Solar Energy Research Institute (SERI), The National University of Malaysia, 43600, Bangi, Selangor, Malaysia; c Department of Physics – Faculty of Natural Sciences, University of Tetovo, Ilindenska pn, 1200, Tetovo, North Macedonia

**Keywords:** CZTS thin films, Stoichiometry, Sol-gel, Spin-coating, Chemicals mixing temperature, Mixing protocol, Band gap, Crystal parameters

## Abstract

In this paper, we examine the impact of the precursor's mixing temperature and mixing protocol on the crystal structure and morphological and optical properties of Cu_2_ZnSnS_4_ (CZTS) thin films. Four samples of CZTS thin films were synthesized with the sol-gel spin coating technique by previously mixing precursors at (a) 150 °C and (b) room temperature (RT), either (i) all at once or (ii) through sequential adding the individual chemicals 30 min apart. SEM-EDX, XRD, Raman and Visible spectroscopy analysis showed that sample 150°C-ST (chemicals mixed at the same time at 150 °C) fulfilled all the theoretical stoichiometric criteria (poor in Cu, rich in Zn) for the high-quality CZTS absorbers. The larger grain size (850 nm) and crystallite size (73.96 nm), lower strain ($0.49\times 10^{-3})$ and band gap $E_{g}=1.44\;eV$ which is closest to the Shockley–Queisser limit for single junction solar cells (1.34 eV).

## 1 Introduction

CZTS (Copper–Zinc–Tin-Sulphur) thin-films are promising materials for low-cost solar cell production, due to their abundance, low cost and non-toxicity. Cu_2_ZnSnS_4_ (CZTS) thin-films are p-type semiconductor with a direct gap that could be tailored within the range of 1.0–1.5 eV and respectable absorption coefficient $(>10^{4}\;cm^{-1}$), making it an absorber layer of choice for PV applications [[1], [2], [3]]. Various synthesizing methods are available for CZTS thin films fabrication, including both vacuum and non-vacuum techniques, such as RF and hybrid (DC and RF) sputtering, spray pyrolysis, chemical vapour deposition, evaporation, co-evaporation, sol-gel and spin-coating [4,5]. Among these, the non-vacuum sol-gel 2-methoxyethanol technique emerged as a promising approach due to its simplicity [2,6], environmental friendliness, low cost, and efficient operability over a wide temperature range [7,8]. The latter can be easily combined with spin coating, allowing film uniformity and thickness control, adaptability of precursor solutions preparation, and the possibility for large-area deposition [8,9]. Besides, the highest reported efficiency achieved so far for with CZTS thin films is 13.6 %, with a deficit in the open-circuit voltage (*V*_oc_) of 297 mV. However, this falls significantly below the theoretical limit of 32 % [10]. The deficit in *V*_oc_ could be attributed to the presence of numerous electronic defects within the CZTS absorber, along with a short minority carrier lifetime, as well as bulk and surface/interface recombination of carriers. In order to mitigate the formation of defects and enhance the performance of the CZTS absorber, a chemical composition with a low copper content (Cu/Sn < 2, Cu/(Zn + Sn) < 0.9) and a high zinc content (Zn/Sn > 1) has been proposed by other authors [11,12]. Spin coating technique was previously used to grow CZTS films by the authors of the present paper in their previous publication [13]. Furthermore, recent research efforts have been primarily focused on post-deposition thermal treatment, to reduce metastable and cluster defects, aiming at improvement of the properties of the CZTS absorber [11]. Little effort has been devoted to the temperature and solution mixing protocol during the synthesis process. There are many reports about synthesizing CZTS from mixed solutions, starting from room temperature conditions, up to 90 °C, as could be found in the following reports concerning the influence of the temperature on spin coated CZTS synthesis: Jiang *at al* [14]. at room temperature, Zakaria et al. 20 min at 25°C [2], Tanaka et al. at 45°C for 1 h [6], Hosseinpour *at al.* at 45°C for 30 min [15], Laghafour at 60°C for 1 h [16], Ahmoum et al. at 90°C for 2 h [17,18], etc. However, to the best of our knowledge, there are no reports on films prepared from precursors being mixed at higher temperatures than 90 °C, using different mixing protocols, which may have an impact on the properties of the CZTS films. The main objective of our investigation is to assess the impact of precursor preparation protocols (chemicals being mixed at two different temperatures – room temperature (RT) and 150°C and under two different regimes: chemicals being mixed all at once (at same time -ST) and chemicals being mixed by adding them to the solution sequentially with 30 min time between (TB), on the CZTS film stoichiometry, Raman binding, crystalline structure and their optical characteristics.

## 2 Experimental

The films were deposited on Na-free glass substrates using a technique we already reported in our previous publication [13]. The glass slides were subjected to the following cleaning protocol: rubbing with deionized water (DIW), followed by an ultrasonic bath in ethanol, acetone and in ethanol again (10 min each bath), and finally by a 20-min bath in DIW. All cleaning procedures were conducted at 80 °C. The substrates were then dried using an $N_{2}$ stream, heated to 120 °C, and placed in an ozone cleaner for 10 min [13]. The chemicals were prepared with the following procedure**. Solution 1** was prepared by mixing the following Sigma-Aldrich reagents: 10ml of 2-methoxyethanol dissolved (1) 0.7M zinc acetate dehydrate (98 % purity), (2) 1.1M of copper acetate dehydrate (98%), (3) 0.6M of tin chloride ($\text{Sn}{\text{Cl}}_{2}∙H_{2}O$, 95 %), and (4) 2.4 M of thiourea (95 % purity). The four different samples, subject to our research, were produced from four different regimes of precursor mixing to obtain Solution 1. **Solution 2** was monoethanolamine (MEA). The first sample was synthesized from a Solution 1 that was prepared by mixing all the reagents at the same time (ST) at room temperature (RT) (Sample RT-ST). The second sample was produced from Solution 1 from the same four chemicals mixed at a same time (ST), but on a hot plate at 150 °C (Sample 150^0^C-ST). The third and the fourth samples were produced from the same precursors as for the first two, only the regime of the mixing Solution 1 was different: the reagents were dissolved in a sequence with 30 min apart (TB-time between). Herein, the third sample was produced from a solution sequentially mixed at RT (Sample RT-TB), while as the fourth sample was made by sequential mixing at 150 °C on a hot plate (Sample 150 °C -TB). After aging for 48 h, the four different “Solutions” were mixed with Solution 2 (MEA) in ratio 1:1. The spin coating process was performed at a substrate rotation speed of 2500rpm for 30s. The samples were then subjected to sulphurisation in a small graphene box furnished with 100mg of S and 20mg of SnS_2_, being finally annealed in $N_{2}$ atmosphere using a tubular furnace (OTF-1200X, MTI-USA) operated at 580° for 30min under a constant base pressure 115mTorr, working pressure 400Torr and a ramp-rate 10°C/min.

To examine the crystalline properties of the four samples of CZTS thin films, X-ray diffraction (XRD) analysis was conducted using a Bruker AXS-D8 advance system equipped with a Cu-Kα source (λ = 1.5406 Å) for 2 Theta angles between 15° to 60°. Furthermore, Raman spectra were recorded with Thermo-Scientific DXR2xi Raman Imaging Microscope. For morphology and composition characterization, a Carl Zeiss Merlin field emission scanning electron microscope (FESEM) at 3 kV was utilized. The grain size of the samples was determined from the SEM-images. The open-source software ImageJ was employed to measure the dimeters of the grains in two dimensions: the longest and shortest diameter of an approximated elliptical shape. The average grain sizes were obtained from the histograms’ normal distribution of the grain size, determined for 50 randomly selected grains from SEM images of choice for each sample. Quantitative and qualitative analysis were performed by Oxford-Instrument INCA 400 energy dispersive X-ray spectroscopy (EDX) at 20 kV operating voltage.

Transmittance spectra were recorded in the range 190 nm–1100 nm with Jasco V-630 UV–Vis Spectrophotometer. The band gap was evaluated using the Tauc-plot, assuming direct transition. The optical band gap (*E*_g_) of each sample was evaluated from the intercept of the extrapolated linear regression model applied on the presentation of (*Ahν)*^2^ versus photon energy plot in the range between 2.5 and 3.5 eV, where *A* is the absorbance of the film, calculated from the transmittance spectra.

## 3 Results and discussion

**Fig. 1:**
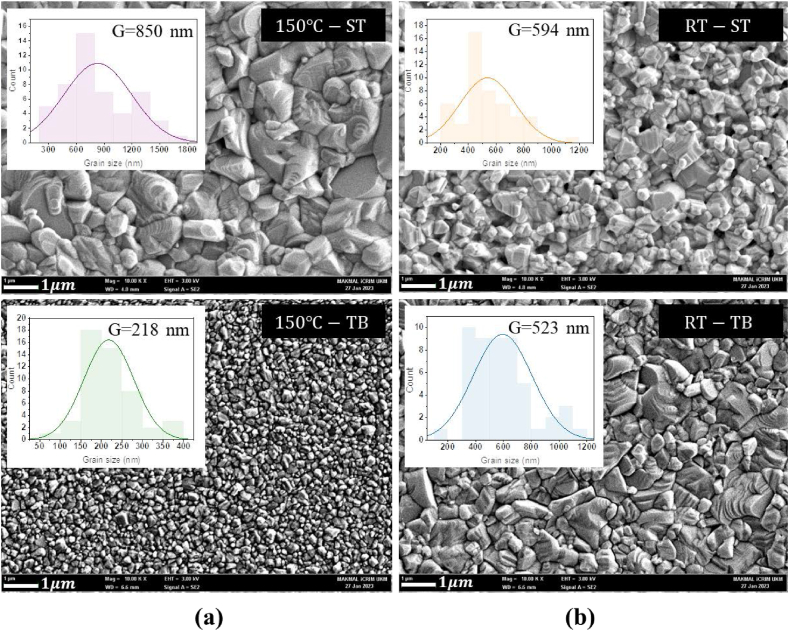
Surface morphology of spin-coated thin films prepared from precursors synthesized from different regimes of mixing precursors: (a) at 150 °C at same time (150 °C -ST) and time between (150 °C -TB) and (b) at room temperature – RT at same time (RT-ST) and time between (RT-TB).

Fig. 1(a) shows the SEM images of spin-coated CZTS films using precursors synthesized at 150 °C (150°C-ST and 150 °C -TB), while as Fig. 1(b) at room temperature RT (RT-ST and RT-TB). In all the SEM images from Fig. 1, grains of different sizes can be observed (as obvious from the grain size histograms implanted within the corresponding SEM images). The average grain size (G) of 850 nm (150^0^C-ST), 218 nm (150^0^C-TB), 523 nm (RT-ST), 594 nm (RT-TB), was evaluated for the four samples (also listed in Table 1). Herein, one may conclude that sample 150°C-ST reveals largest grains, while 150°C-TB the smallest average grain size, implying that the regime of precursor mixing impacts the film's grain size profoundly (four times).

**Table 1:** Grain size ‘G’, CZTS thin films stoichiometry (molar ratios, derived from atomic percentage of FESEM-EDX), lattice parameters ‘a’, ‘c’,‘$d_{112}$’, Volume ‘V’, Micro-strain ‘ε’, Crystallite size ‘*D’* evaluated from XRD patterns and band gap energy ‘*E*_g_’ of four samples, from precursors mixed at 150 °C or at room temperature (RT), mixed at the same time (ST) or with time between (TB).

	G [nm]	Cu/Sn	Cu/(Zn + Sn)	Zn/Sn	S/metals	a(Å)	c(Å)	$d_{112}\left(Å\right)$	$V\;\left({Å}^{3}\right)$	$\varepsilon \left(\times 10^{-3}\right)$	*D* (nm)	*E*_g_ [eV]
150°C-ST	850	1.47	0.68	1.17	1.20	5.42	10.81	3.126	317.58	0.49	73.96	1.44
RT-ST	523	1.62	0.83	0.94	1.27	5.42	10.81	3.126	317.58	0.59	61.71	1.49
150°C-TB	218	1.08	0.68	0.58	1.48	5.42	10.79	3.123	316.72	0.64	56.44	1.46
RT-TB	594	1.89	0.72	0.61	1.43	5.42	10.80	3.125	317.26	0.72	50.17	1.57

**Fig. 2:**
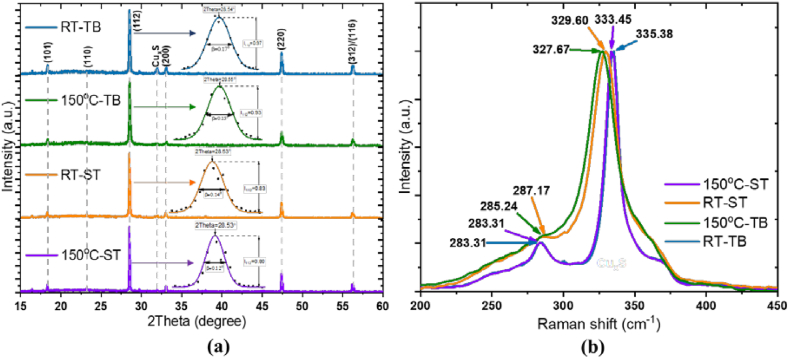
(a) XRD diffractograms with (112) peak analysis within, and (b) Raman spectra of the four CZTS spin-coated thin films from precursors mixed at 150 °C or at room temperature (RT), mixed at the same time (ST) or with time between (TB).

Fig. 2(a), shows the X-ray diffraction patterns of the four CZTS spin-coated films, all revealing a preferential crystallographic orientation along the (112)-direction at 2Theta 28.53°. The other pronounced diffraction peaks are evident along the (101), (110), (200), (220) and (312)/(116) planes at 2Theta angles 18.31°, 23.20°, 33.03°, 47.40° and 56.15°, respectively. These results suggest a typical kesterite crystal phase of CZTS (JCPDS 26–0575) [19,20]. Other negligible peaks are also evident, which can be related to the presence of secondary phases, mainly from Cu_2-x_S and ZnS [21]. In addition, the (112)-peak fitting procedure with a Gaussian function was performed the full width at the half maximum -FWHM (β), and the crystallite size (*d*). Evidently, the Samples 150°C-ST and RT-ST have lower values of 2Theta (28.53°) and β, indicating larger interatomic spacing and larger crystallite size, while at samples 150°C-TB and RT-TB the main (112) peaks are identified to be slightly more intense and wider, indicating better crystallinity and increased structural disorder. Herein one can conclude that the larger values of the strain correspond to smaller average value of the crystallite size (wider peaks). The lattice parameters (***a*** and ***c***) of CZTS thin films are shown in Table 1, being calculated from combining Bragg's Law (Eq. (1)), Debye Scherrer's formula (Eq. 2) and taking in consideration Millers indices (hkl) for tetragonal body-centered kesterite structure (Eq. (3)) [20,22].

(Eq. 1) λ=2dsinθ

(Eq. 2) $$D=\frac{0.94\lambda }{\beta \;\cos\;\theta }$$

(Eq. 3) $$\frac{1}{d^{2}}=\frac{h^{2}+k^{2}}{a^{2}}+\frac{l^{2}}{c^{2}}$$

where D is the crystallite size, β indicates FWHM of the X-ray diffraction peak in radians, λ is the wavelength of the X-ray, d the interplanar spacing and θ is the Bragg's diffraction angle. Herein, the microstrain (ε) values of CZTS thin films, were obtained using Eq. (4).

(Eq. 4) $$\varepsilon =\frac{\beta \;\cot\;\theta }{4}$$

The Raman spectra presented in Fig. 2 (b) reveal two distinct peaks for all samples attributed to A1 and A2 vibration modes: one at 283.31 cm^−1^ (found for 150^0^C-ST and RT-TB films), 285.24 cm^−1^ (150^0^C-TB) and 287.17 cm^−1^ (RT-ST) which arises from the vibration of Cu atoms in the CZTS crystal structure (known to appear around 287 cm^−1^) [23,24], and the others at 327.67 cm^−1^ (150^0^C-TB), 329.60 cm^−1^ (RT-ST), 333.45 cm^−1^ (150^0^C-ST) and 335.38 cm^−1^ (RT-TB), related to the presence of Cu, Zn, and Sn in the CZTS film [16,24].

Table 1 summarizes the results of the elemental molar content (relevant metallic elements ratios) obtained by FESEM-EDX. Based on the reports regarding the high-quality criteria for CZTS absorbers, a Cu-poor composition (Cu/Sn < 2, Cu/(Zn + Sn) < 0.9) is recommended and a Zn-rich content (Zn/Sn > 1) [11,12] is considered favourable for the PV efficiency. Results presented in Table 1 show that only Sample 150°C-ST satisfies all the recommended criteria Cu/Sn = 1.47 < 2; Cu/(Zn + Sn) = 0.68 < 0.9; and Zn/Sn = 1.17 > 1. Herein, slightly different Zn/Sn ratios are observed in the stoichiometry of the other samples. Also, the table includes information of crystalline parameters, strain and crystallite size (*D)* obtained from the diffractograms of the respective samples.

**Fig. 3:**
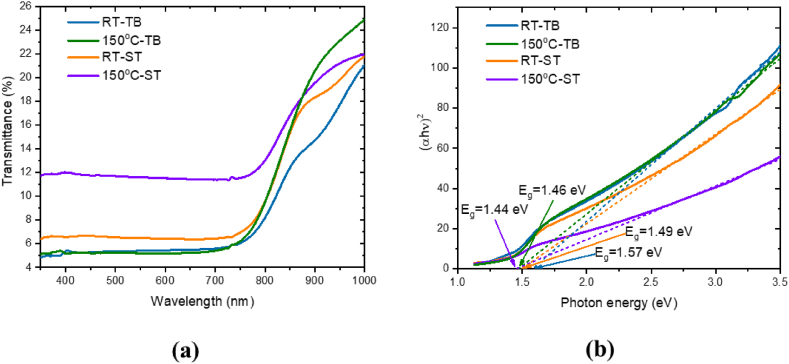
(a) Transmittance spectra and (b) Tauc plot of the four CZTS samples of CZTS spin-coated thin films from precursors mixed at 150 °C or at room temperature (RT), mixed at the same time (ST) or with time between (TB).

Finally, the transmittance spectra presented in Fig. 3(a) show a good absorption of all films in the visible part of the spectrum. The Tauc plot in Fig. 3(b) was used to evaluate the band gap (*E*_g_) of each film. As evident, the Sample RT-TB shows the widest gap (E_g_ = 1.57 eV) while as the Sample 150^0^C-ST the narrowest (*E*_*g*_ = 1.44 eV). Again, the Sample 150^0^C-ST shows the value of the band gap that is considered to be closest to the direct gap of GaAs (1.42 eV) [25] and also closest to the Shockley–Queisser limit for single junction solar cells (1.34 eV) at solar spectrum AM1.5 [26].

## 4 Conclusion

Four spin-coated samples of CZTS thin films were synthesized by mixing the precursors under four different regimes: either reagents mixed at the same time (ST) for 2 h, or with 30 min time between (TB) at two different temperatures, either at RT or 150 °C, followed by annealing process in $N_{2}$ atmosphere. The results showed that the precursor mixing (temperature and regime of mixing) impacts on the spin-coated CZTS film stoichiometry, Raman binding, crystalline structure and optical characteristics. It was observed that the CZTS thin films prepared by mixing precursors at 150 °C all at once (Sample 150°C-ST) fulfils all the theoretical stoichiometric criteria (poor in Cu and rich in Zn) for the high-quality CZTS absorbers, also showing greater values of the grain size (850 nm) and crystallite size (73.96 nm), lower values of strain ($0.49\times 10^{-3})$, good absorption and very suitable band gap for better solar light absorption ($E_{g}=1.44\;eV)$. Based on this, we conclude that CZTS sol-gel spin coated thin films, obtained from precursors synthesized at 150 °C from reagents mixed all at once are suitable for photovoltaic applications due to the formation of greater grains and crystallites, both providing larger charge mobility and reduced defects that induce higher recombination of the photogenerated carriers at the grain boundaries. Also, for the same films the lower strain provides better mechanical stability and hence durability of the CZTS films.

## Data availability

The raw data from (1) Transmittance spectra data, (2) XRD patterns data are deposited at public repository of the Ss. Cyril and Methodius University in Skopje, North Macedonia and could be accesses using the following link: https://repository.ukim.mk/simple-search?query=Mimoza+Ristova+CZTS.

## CRediT authorship contribution statement

**R. Aliti:** Writing – review & editing, Writing – original draft, Methodology, Investigation, Funding acquisition, Formal analysis, Data curation, Conceptualization. **Y. Putthisigamany:** Methodology, Investigation. **P. Chelvanathan:** Project administration, Methodology, Investigation, Conceptualization. **M. Ristova:** Writing – review & editing, Writing – original draft, Validation, Supervision, Methodology, Formal analysis, Conceptualization.

## Declaration of competing interest

The authors declare that they have no known competing financial interests or personal relationships that could have appeared to influence the work reported in this paper.
